# A Ni(OH)_2_/Ti^3+^–titania photocatalyst produced *via* wet fine-bead milling and accelerated hydrogen production from seawater

**DOI:** 10.1039/d6ra05520f

**Published:** 2026-09-07

**Authors:** Shoichi Somekawa, Sayaka Yanagida, Naoki Tachibana, Hiroaki Imai, Shigeru Nakazawa

**Affiliations:** a Tokyo Metropolitan Industrial Technology Research Institute 2-4-10 Aomi, Koto-ku Tokyo 135-0064 Japan somekawa.shouichi@iri-tokyo.jp; b Department of Applied Chemistry, Faculty of Science and Technology, Keio University 3-14-1 Hiyoshi, Kohoku-ku Yokohama 223-8522 Japan; c Photogen Co., Ltd 13F-1-11-1 Marunouchi Chiyoda-ku Tokyo 100-6208 Japan

## Abstract

Ni(OH)_2_/Ti^3+^-doped titania prepared by wet fine-bead milling exhibited a hydrogen production rate 45 times higher than that of pristine titania under UV and visible light in the presence of a sacrificial agent. Adding artificial seawater to distilled water containing 0.005 vol% ethanol increased the rate of H_2_ production fivefold, suggesting that the oxidation of chloride ions was promoted.

## Abstract 2

Ni(OH)_2_/Ti^3+^‑doped titania prepared by wet fine‑bead milling in ethanol showed a 45‑fold increase in photocatalytic activity relative to pristine titania. Hydrogen evolution was promoted by the synergistic effect of trace ethanol and seawater.

## Graphical abstract

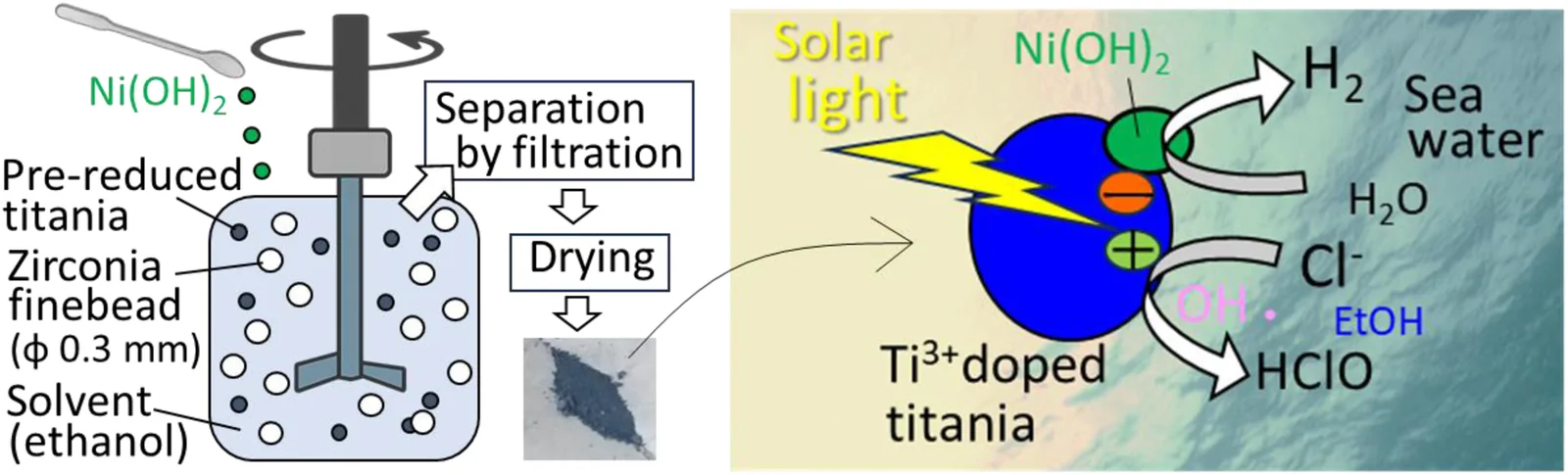

## 1. Introduction

The combustion of hydrogen produces only water as a byproduct, and so hydrogen has received significant attention as a clean energy carrier, with broad applicability in fuel cells and co-firing systems that can substantially reduce CO_2_ emissions. Even so, approximately 95% of the hydrogen currently in use is derived from fossil fuels, such that its use is not entirely carbon neutral. Solar-driven water splitting represents an ideal alternative approach to the generation of hydrogen as a sustainable, carbon-free energy source. In this regard, the use of seawater (which comprises approximately 98% of the planet's water) would be highly desirable. Although examples of photocatalytic seawater splitting have been reported,^1–11^ practical applications remain challenging because such processes tend to exhibit low efficiency and a lack of robustness. The latter is caused by chlorine-related by-products such as hypochlorous acid, resulting from the high concentration of salts (approximately 3.4 wt%) in seawater.

Titania (TiO_2_) has been used as a photocatalyst ever since the initial discovery of the Honda-Fujishima effect.^12^ This compound is commonly employed for environmental purification and self-cleaning applications,^13–15^ because it is non-toxic, stable, readily available and inexpensive. Unfortunately, titania absorbs only ultraviolet (UV) light and shows poor performance when used to produce hydrogen and oxygen *via* the decomposition of water.

Beginning in 2009, researchers began to consider the introduction of Ti^3+^ into titania for the purpose of providing a visible light response and improving photocatalytic activity.^16–23^ Recently, the author developed a means of doping Ti^3+^ into titania in a stable manner while maintaining a high specific surface area and suppressing crystal collapse, based on a wet bead-milling method using fine zirconia beads and ethanol.^24^

In the present study, powdered chemical compounds such as Ni(OH)_2_ were incorporated during the bead-milling process used for Ti^3+^ doping. The effects of this modification on the photocatalytic activity of the product when used with a sacrificial agent under UV and visible light were then assessed. This material was also applied to the splitting of seawater for hydrogen production.

## 2. Experimental

Reduced titania (Photogen Co.) was subjected to wet bead-milling in ethanol using a batch-type mill equipped with 0.3 mm zirconia beads (Fig. 1(a)). The photocatalytic activity of the resulting product was subsequently evaluated by dispersing 0.01 g of the photocatalysts in 20 mL of an aqueous solution in a 50 mL cylindrical reactor, followed by irradiation with UV and visible light under a 7 mL min^−1^ Ar-flow (Fig. 1(b)). The hydrogen evolution rate was continuously monitored (See Fig. S1–S3 in the SI for experimental details).

**Fig. 1:**
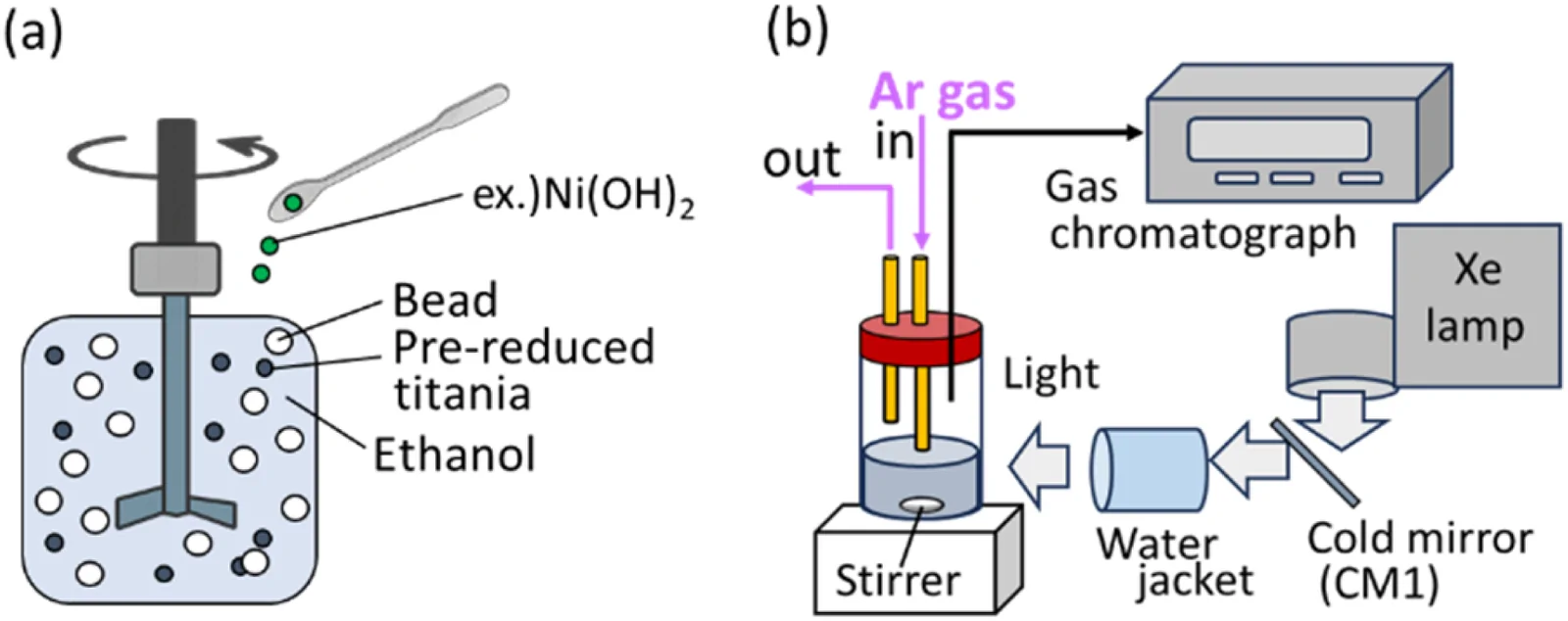
Diagrams of experimental systems used for (a) wet-type bead-milling and (b) assessment of hydrogen production rate.

## 3. Results and discussion

During initial trials, the hydrogen production rate was assessed while using an aqueous solution containing 20% as a sacrificial reagent under UV and visible light.

Fig. 2(a) plots hydrogen production rates over time for such trials with Ni(OH)_2_ (5 wt%)/Ti^3+^-doped titania, Ti^3+^-doped titania, pre-reduced titania and pristine titania (ST01). The specific surface area of the pristine titania was 289 m^2^ g^−1^ and this value was reduced to 51 m^2^ g^−1^ after the high-temperature pre-reduction step prior to bead-milling. Note that, in order to dope the titania lattice with Ti^3+^ ions *via* bead-milling in a stable manner, it was necessary to introduce a sufficient degree of oxygen deficiency in the stage prior to the bead-milling process.^18^

**Fig. 2:**
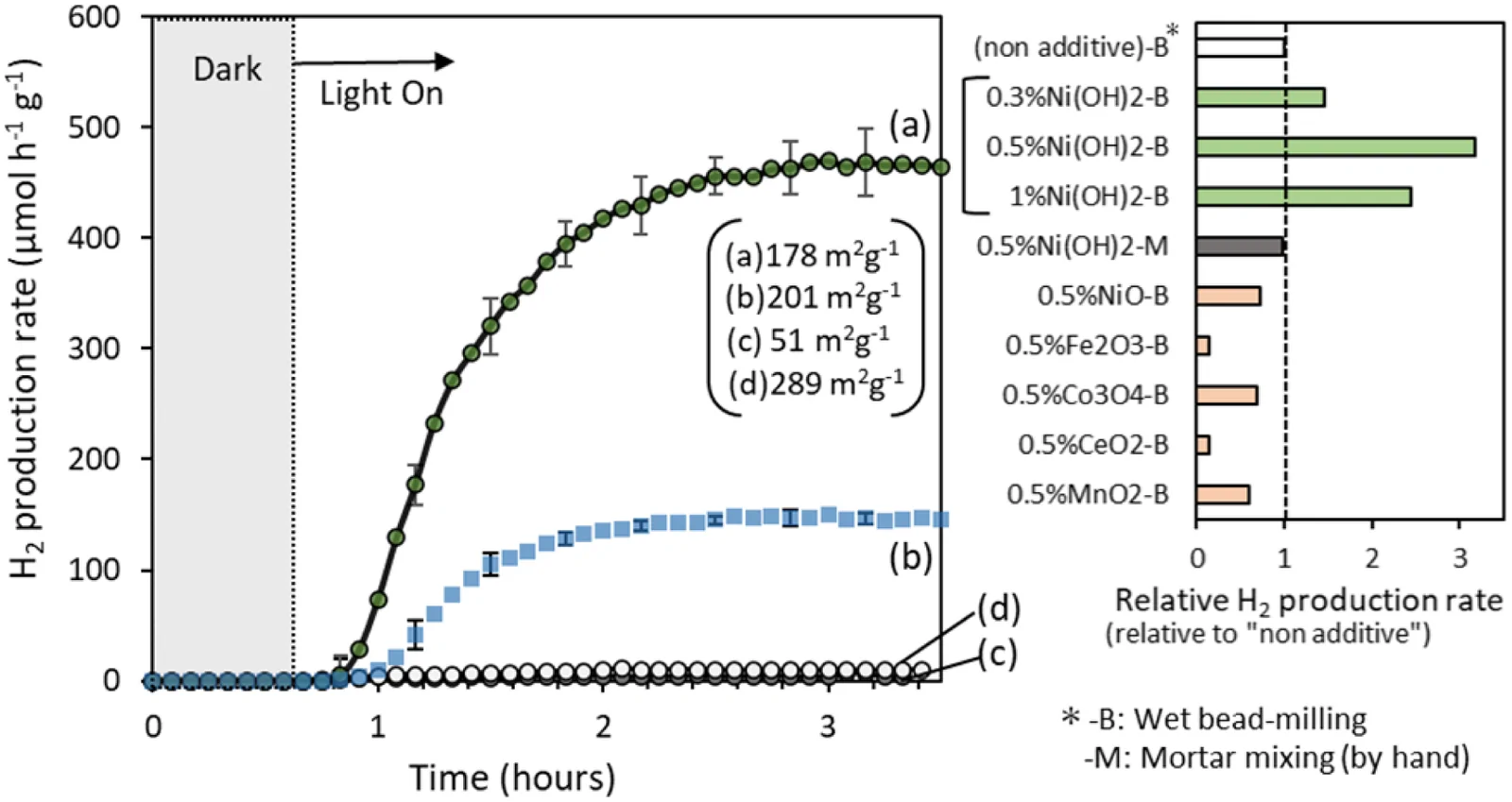
(a) Hydrogen production rates obtained using (i) Ni(OH)_2_ (0.5 wt%) on Ti^3+^-doped titania, (ii) Ti^3+^-doped titania, (iii) pre-reduced titania, and (iv) pristine titania (ST01). Numerical values indicate surface areas of the photocatalyst. (b) Relative H_2_ production rates, defined as: (H_2_ production rate with additive)/(H_2_ production rate without additive). Error bars represent the standard deviation (*n* = 3). Experimental conditions: photocatalyst 0.01 g; suspension 20 mL (20 vol% ethanol); irradiation by a 450 W xenon lamp with CM1 cutoff filter (range: *ca.* 300–1200 nm).

For this reason, the pristine titania was reduced prior to milling. Subsequent milling with 0.3 mm beads in ethanol increased the surface area to 201 m^2^ g^−1^ without an additive and to 178 m^2^ g^−1^ with 0.5 wt% Ni(OH)_2_. Doping with Ti^3+^ was found to greatly increase the hydrogen production rate, to approximately 15 times that for the pristine titania. The addition of Ni(OH)_2_ during the milling process further increased the activity, by *a* factor of approximately 45. Fig. 2(b) summarizes the relative hydrogen production rates normalized to that obtained from a titania specimen without additives. The optimal Ni(OH)_2_ loading in conjunction with wet bead-milling was evidently 0.5 wt%. Interestingly, simple manual mixing of Ni(OH)_2_ with the titania at a 0.5 wt% ratio using a mortar and pestle did not show any increase in the catalytic activity. Adding NiO and other metal oxides at the same ratio (0.5 wt%), on the other hand, decreased the activity. The bead-milling process is considered to promote the high dispersion of Ni(OH)_2_ on the titania surface through bonding with the surface hydroxyl groups.^25^ Ni(OH)_2_ then acts as a co-catalyst for hydrogen evolution by trapping electrons at the hetero-interface and promoting the charge separation,^26^ thereby enhancing the photocatalytic activity.

The field-emission scanning electron microscopy (FE-SEM) image in Fig. 3(a) and the corresponding elemental maps in Fig. 3(b)–(d) establish that particles of a Ni compound were dispersed over the titania particles. This Ni compound was identified as Ni(OH)_2_ by X-ray absorption near-edge structure (XANES) analysis (Fig. 3(e)). The white-line feature arises from the 1s → 4p transition and is influenced by the local coordination environment and the oxidation state of Ni.^27^ The absorption spectra, XRD patterns, and XPS patterns of the sample are shown in Fig. S4–S6 in the SI.

**Fig. 3:**
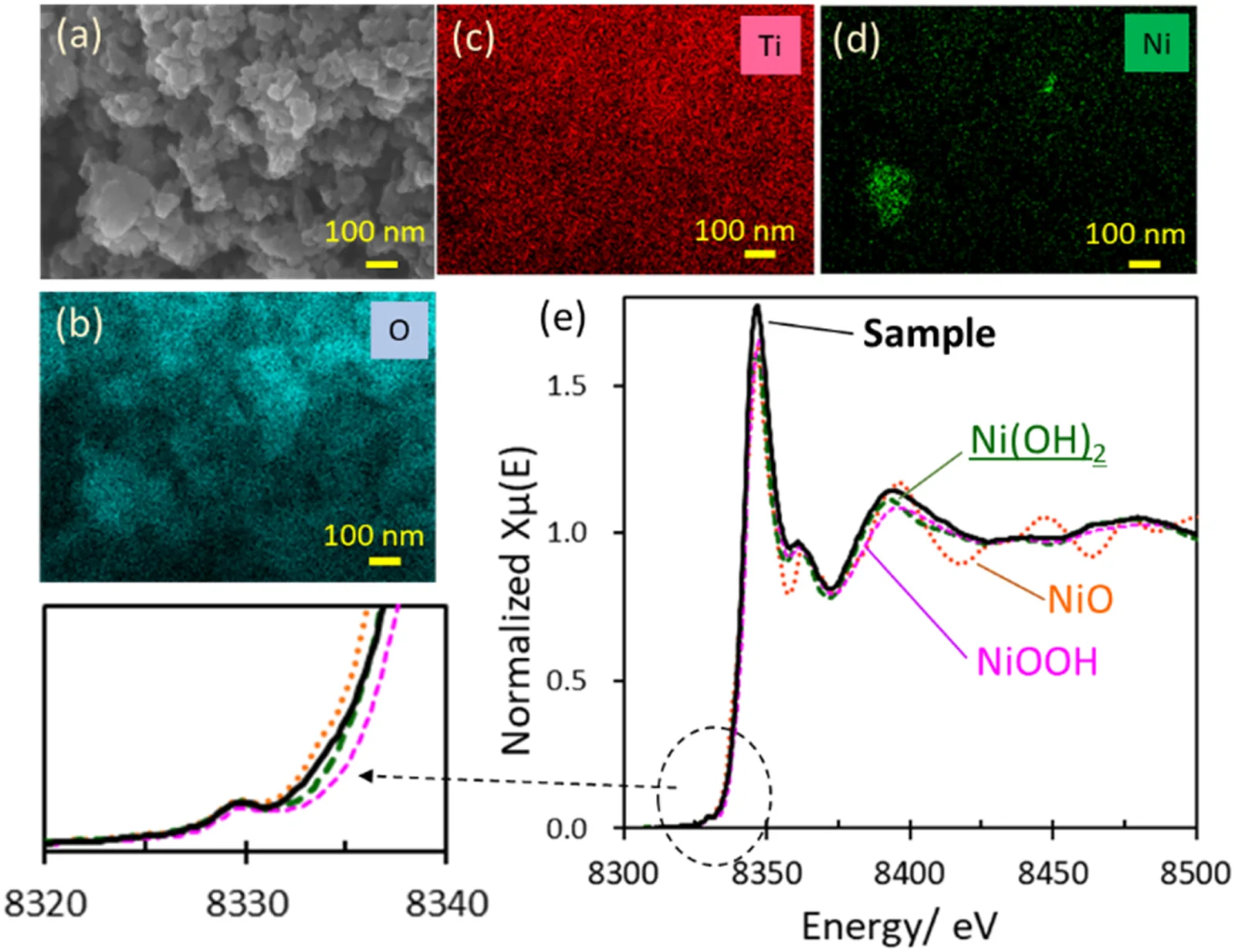
FE-SEM images (a) (5 kV, tungsten pre-coating), the elemental maps ((b) O, (c) Ti, (d) Ni) and XAFS spectra (e) of Ni in the Ni(OH)_2_/Ti^3+^-doped titania and some reference samples.

The wet-milled Ni(OH)_2_/Ti^3+^-doped titania photocatalyst containing 0.5 wt% Ni(OH)_2_ [labelled “0.5%Ni(OH)_2_-Bz” in Fig. 2(b)] was subsequently applied to seawater splitting.

Fig. 4 shows the effect of adding artificial seawater (at ten times the salt concentration of actual seawater) to aqueous solutions both with and without 0.005 vol% ethanol on the hydrogen production rate. Note that only a minimal amount of ethanol was used because higher concentrations were found to inhibit seawater splitting.

**Fig. 4:**
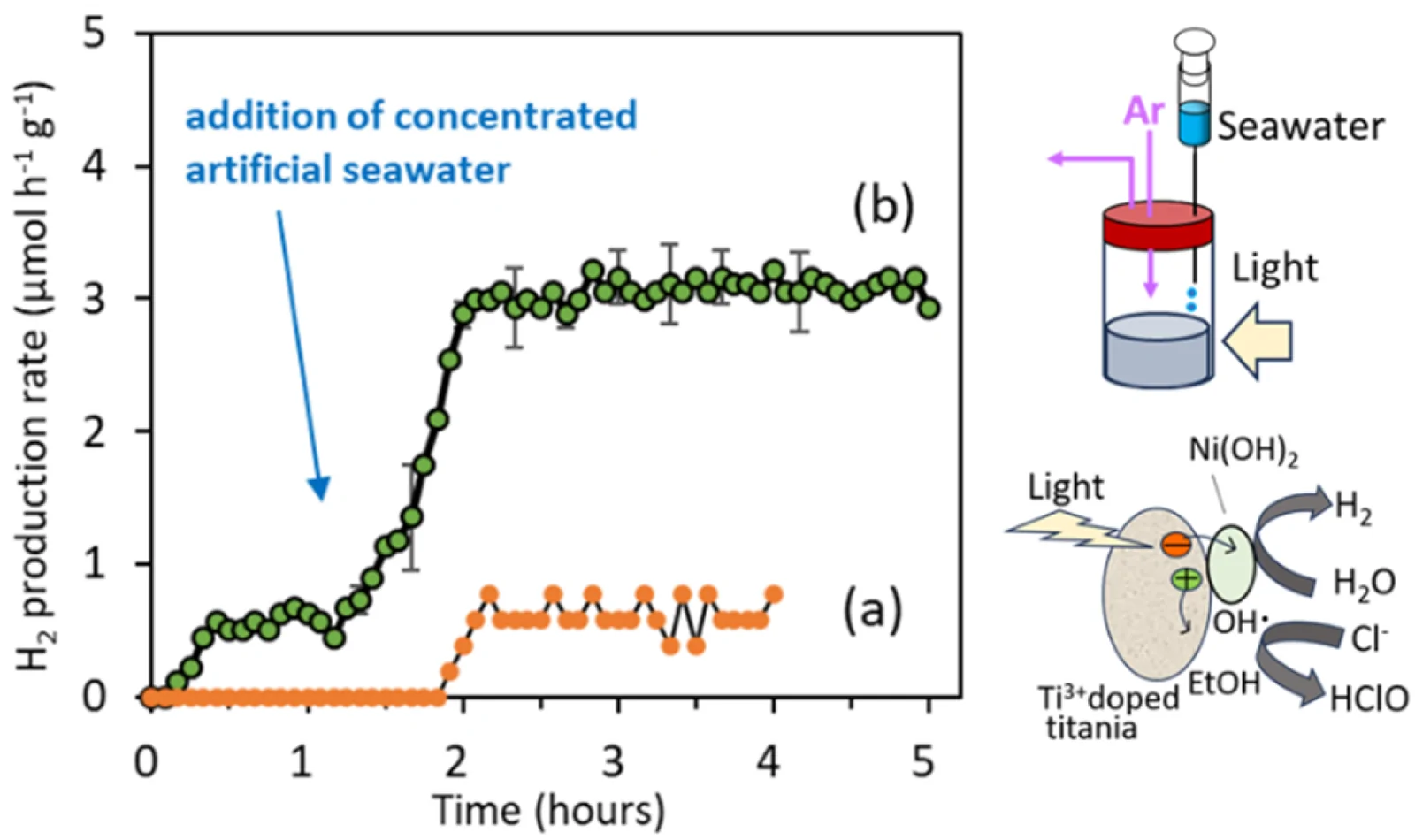
Effect of adding concentrated artificial seawater (a) without and (b) with trace amount of ethanol (0.005 vol%) on hydrogen production rate. Error bars represent the standard deviation (*n* = 3). Experimental conditions: Ni(OH)_2_/Ti^3+^-doped titania 0.01 g, distilled water 20 mL, 450 W xenon lamp with CM1 cutoff filter. Insert in the upper right: experimental setup. Insert in the lower right: proposed reaction pathway.

The right side of Fig. 4 shows diagrams of the experimental setup and the proposed reaction pathway. Photogenerated electrons (indicated by the symbol −) and holes (indicated by the symbol +) drive the redox reactions. Specifically, electrons transferred to Ni sites promote hydrogen evolution,^26^ whereas holes generate OH radicals.^28^ These radicals react with ethanol molecules but also oxidize chloride ions to produce hypochlorous acid. The plot labelled (a) in Fig. 4 shows that no hydrogen was obtained when the photocatalyst was irradiated in pure distilled water. Following the addition of 1 mL of the 10× concentrated artificial seawater (the final concentration after addition is approximately 1.7 wt%, about half that of natural seawater), a small amount of hydrogen was produced, indicating that the chloride ions acted as a sacrificial reagent. The plot labelled (b) indicates the hydrogen production rate in the presence of a small amount of ethanol acting as a sacrificial donor. This solution produced a low level of hydrogen prior to the addition of concentrated artificial seawater. Incorporating the seawater increased the hydrogen production rate five-fold, demonstrating a synergistic effect between the ethanol and the chloride ions. Analysis using *N*,*N*-diethyl-*p*-phenylenediamine^29^ showed a free chlorine concentration of 1 ppm resulting from the formation of hypochlorous acid. This finding suggests that chloride oxidation was promoted in the presence of a trace of ethanol. Note that the free chlorine in the solution was unstable. The reaction rate remained substantially constant, and no decrease in catalytic activity was observed under the conditions. (Effects of the amount of concentrated artificial seawater added and the ethanol concentration are shown in Fig. S7 in the SI; the XPS spectra before and after the reaction are shown in Fig. S8 in the SI; the results of the control experiment using Na_2_SO_4_ instead of concentrated artificial seawater are presented in Fig. S9 in the SI; and a hydrogen-production experiment using real seawater to confirm catalyst stability is shown in Fig. S10 in the SI.)

## 4. Conclusion

In this study, titania was doped with Ti^3+^ in a stable manner *via* a wet fine-bead milling method. The effects of adding various oxides during the milling process on the photocatalytic activity were investigated. The incorporation of 5 wt% Ni(OH)_2_ during the milling step as a means of ensuring doping with Ti^3+^ in a stable manner markedly enhanced hydrogen evolution from water containing ethanol as a sacrificial reagent in response to UV-visible radiation. The performance of the material was increased by *a* factor of approximately 45 compared with that of pristine titania. The addition of concentrated artificial seawater to a system comprising Ni(OH)_2_/Ti^3+^-doped titania with a trace amount of ethanol increased the hydrogen production fivefold, suggesting the accelerated oxidation of chloride ions to hypochlorous acid. These findings are expected to promote the design of practical seawater splitting systems for hydrogen production using inexpensive, stable photocatalysts.

## Conflicts of interest

There are no conflicts to declare.

## Supplementary Material

RA-016-D6RA05520F-s001

## Data Availability

The data that support the findings of this study are available from the corresponding author upon reasonable request. Supplementary information (SI): (1) preparation of a Ni(OH)_2_/Ti^3+^-doped titania photocatalyst. (2) Characterization. (3) Measurement of photocatalytic activity. (4) XRD analysis. (5) Absorption spectra and visible light responsiveness (under UV light irradiation). (6) XPS analysis. (7) Effects of the amount of concentrated artificial seawater added and the ethanol concentration. (8) Comparison of the XPS spectra (Ti and Ni) of the samples before and after the reaction (Fig. 4 in the main text). (9) Addition of 10 wt% Na_2_SO_4_ solution instead of 10 wt% artificial seawater. (10) Application to real seawater splitting in Tokyo Bay. (11) References. See DOI: https://doi.org/10.1039/d6ra05520f.
