# Supplementary material for: A Ni(OH)2/Ti3+–titania photocatalyst produced via wet fine-bead milling and accelerated hydrogen production from seawater
Source: RSC Adv. 2026 Sep 7;16(43):53252–5. doi: 10.1039/d6ra05520f (PMC13549190; doi:10.1039/d6ra05520f)
Supplement: Supplementary file 1 [file RA-016-D6RA05520F-s001.pdf]

Supplementary Information (SI) for RSC Advances.
This journal is © The Royal Society of Chemistry 2026

Supporting Information

A $Ni(OH)_2/Ti^{3+}$–Titania Photocatalyst produced via Wet Fine-bead Milling and Accelerated Hydrogen Production from Seawater

Shoichi Somekawa[a*], Sayaka Yanagida[a], Naoki Tachibana[a], Hiroaki Imai[b] and Shigeru Nakazawa[c]

a Tokyo Metropolitan Industrial Technology Research Institute, 2-4-10 Aomi, Koto-ku, Tokyo, 135-0064, Japan.
b Department of Applied Chemistry, Faculty of Science and Technology, Keio University, 3-14-1 Hiyoshi, Kohoku-ku, Yokohama, 223-8522, Japan.
c Photogen Co., Ltd., 13F-1-11-1 Marunouchi Chiyoda-ku, Tokyo, 100-6208, Japan.

Corresponding author
*E-mail: Somekawa.shouichi@iri-tokyo.jp

(1) Preparation of a $Ni(OH)_2/Ti^{3+}$-doped titania photocatalyst.

Titania (TiO_2, ST01, ≥ 99.9%, anatase, Ishihara Sangyo Co., ltd) was used as the raw material. This material was calcined at 600 °C for 3 h under reducing conditions to introduce a suitable concentration of oxygen vacancies into the titania lattice prior to the wet bead-milling (the pre-reduced sample was provided by Photogen Co., Ltd). The wet bead-milling was performed with a batch-type bead mill (Labstar HFM02, Ashizawa Finetech Ltd.) using zirconia beads with a diameter of 0.3 mm (Nikkato Corp.). A slurry with a bead packing proportion of approximately 50% was prepared by mixing 300 g of zirconia beads with 0.43 g of the pre-reduced titanium dioxide in 130 mL of ethanol (C_2H_5OH, 99.5%, FUJIFILM Wako Pure Chemical Corporation).

0.0022 g of Nickel (II) hydroxide ($Ni(OH)_2$, 99.5%, FUJIFILM Wako Pure Chemical Corporation) was added to the system. All chemicals and reagents were used without further purification.

The slurry was subsequently milled at 1910 rpm for 3 h, after which the beads were removed by filtration and the product was evaporated to dryness in air to obtain a powder. The system's overview is shown in Figure S1.

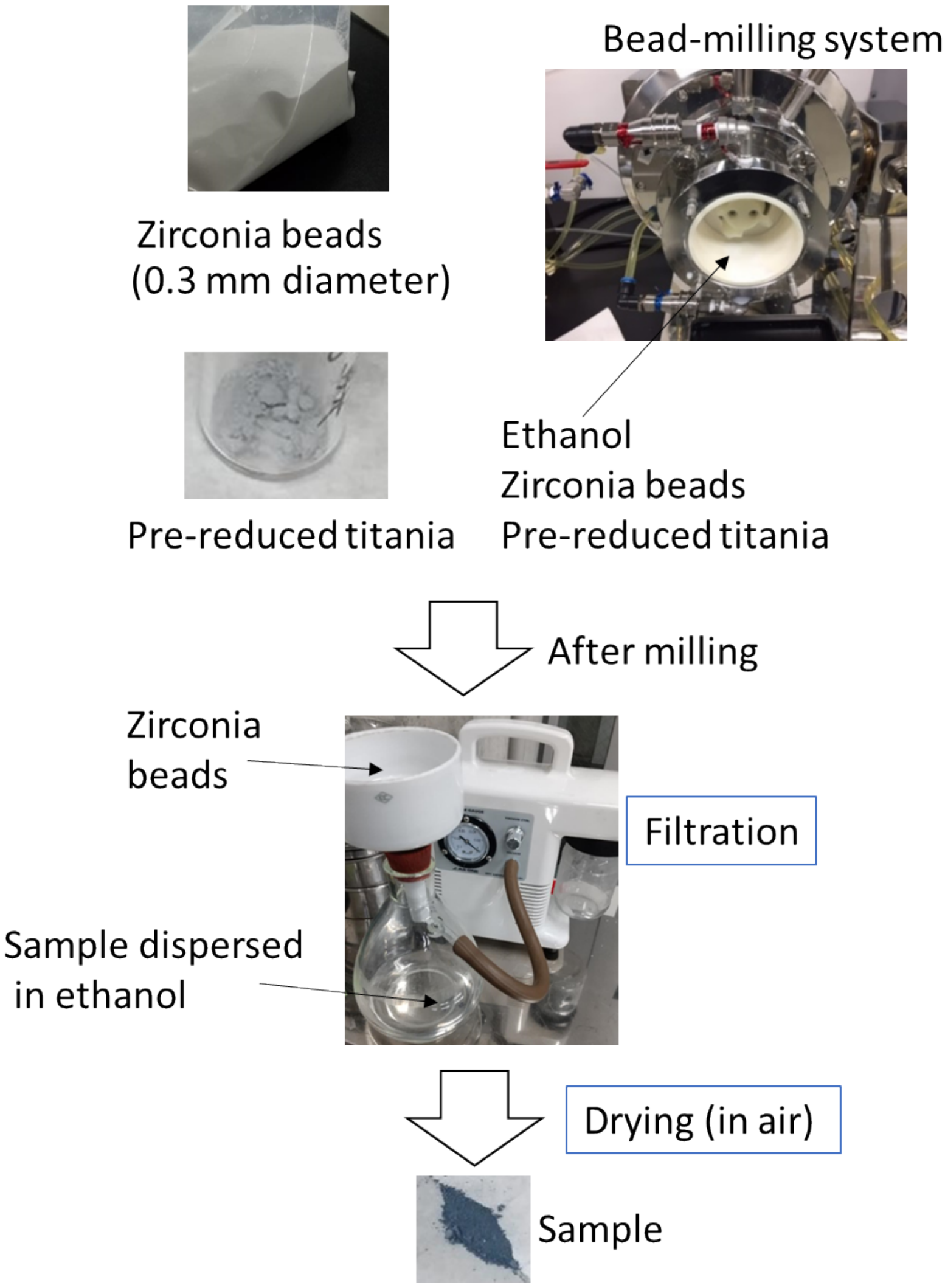

Figure S1. Overview of the bead-milling system.

(2) Characterization

Brunauer-Emmett-Teller (BET) [1] specific surface areas were measured at 77 K using N_2 gas with a BELSORP-max (MicrotracBEL Corp.) apparatus. The sample was outgassed at 120 °C for 3 h prior to the measurements. Field emission scanning electron microscopy (FE-SEM) images were obtained using a SU8600 (EDX: Ultim Max 100) (Hitachi Corp.) after coating samples with tungsten. Ni K-edge X-ray absorption fine structure (XAFS) data were acquired in transmission mode using Si (111) double-crystal monochromators and seven channels silicon drift detector at the hard X-ray HAFS1 beamline within the Aichi Synchrotron Radiation Center. UV–visible diffuse reflectance spectra of the samples were acquired using a V-670 instrument (JASCO Corp.). X-ray diffraction (XRD) patterns were recorded with a Smartlab diffractometer (Rigaku Corp.) using Cu Kα radiation (50 kV, 40 mA).

(3) Measurement of photocatalytic activity

The photocatalytic activity of each specimen was evaluated by placing a 0.01 g sample in a cylindrical 50 mL flask containing 20 mL of a 20 vol% aqueous ethanol solution under a continuous 7 mL/min flow of Ar gas (the photographs of the experimental setup are shown in Figure S2). Both UV and visible light were provided by a xenon lamp (300W, CX-05E, Eagle Engineering Corp.) with a cold mirror (CM1). Hydrogen gas generated during each reaction was quantitatively analyzed using a gas chromatograph (GC, 3000A, Agilent Technologies Corp.) equipped with a molecular sieve column and a thermal conductivity detector, employing Ar as the carrier gas.

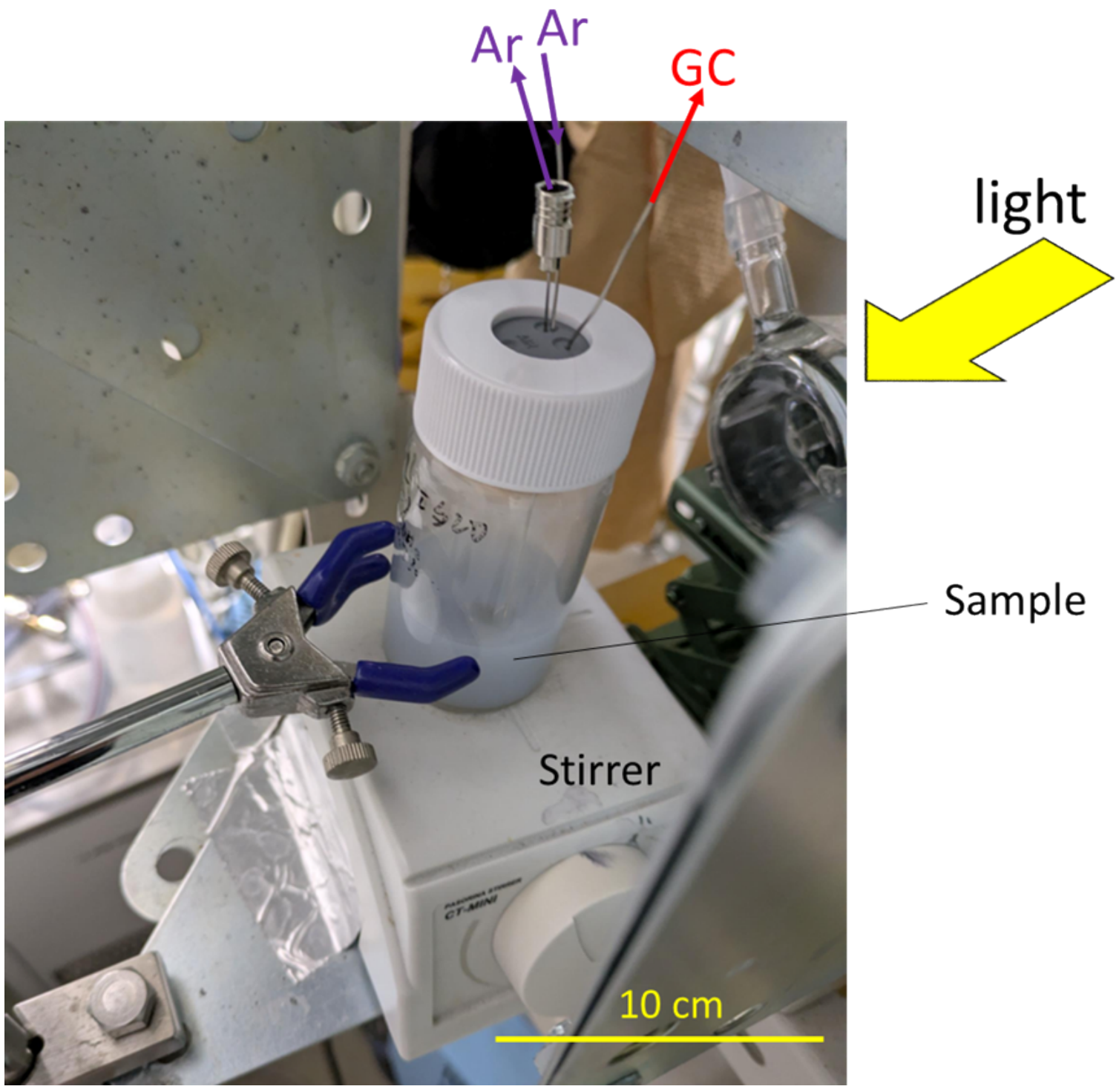

Figure S2. Photographs of the experimental setup for photocatalyst evaluation.

Figure S3 illustrates the wavelength range of the light source used. Both UV and visible light (with a UV power density (300–400 nm) of 5 mW/cm^2) were provided by a xenon lamp (CX-05E,Eagle Engineering Corp.) with a cold mirror (CM1).

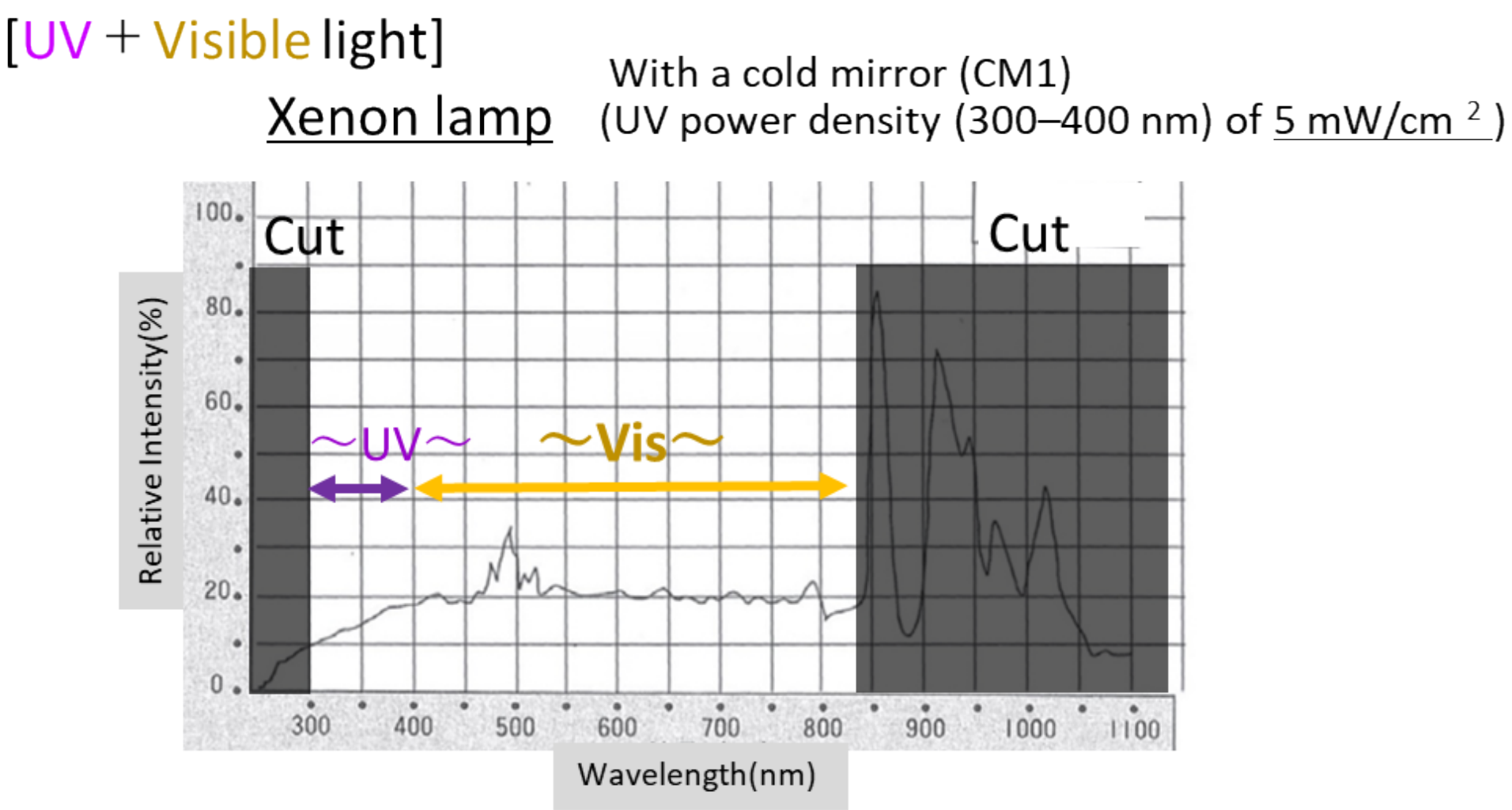

Figure S3 Emission spectra of the UV-visible light sources.

(4) XRD analysis

Figure S4 shows the XRD patterns of each sample.

In the sample (pre-reduced titania) prepared by reducing the raw titania (pristine titania), the anatase peak increased due to sintering (with a slight appearance of rutile peaks). In the wet bead-milled sample (Ti^{3+}-doped titania), the anatase peak intensity decreased due to the milling process (the rutile peak almost completely disappeared). The anatase peak intensity of the sample composited with $Ni(OH)_2$ ($Ni(OH)_2/Ti^{3+}$-doped titania) was approximately the same as that of the sample without the $Ni(OH)_2$ composite (Ti^{3+}-doped titania). No effect on the crystal structure resulting from the $Ni(OH)_2$ composite was observed. Since the anatase peak intensities of Ti^{3+}-doped titania and $Ni(OH)_2/Ti^{3+}$-doped titania were comparable to that of pristine titania, it is considered that the bead-milling process performed under the current conditions did not have a disadvantageous effect on the photocatalytic activity.

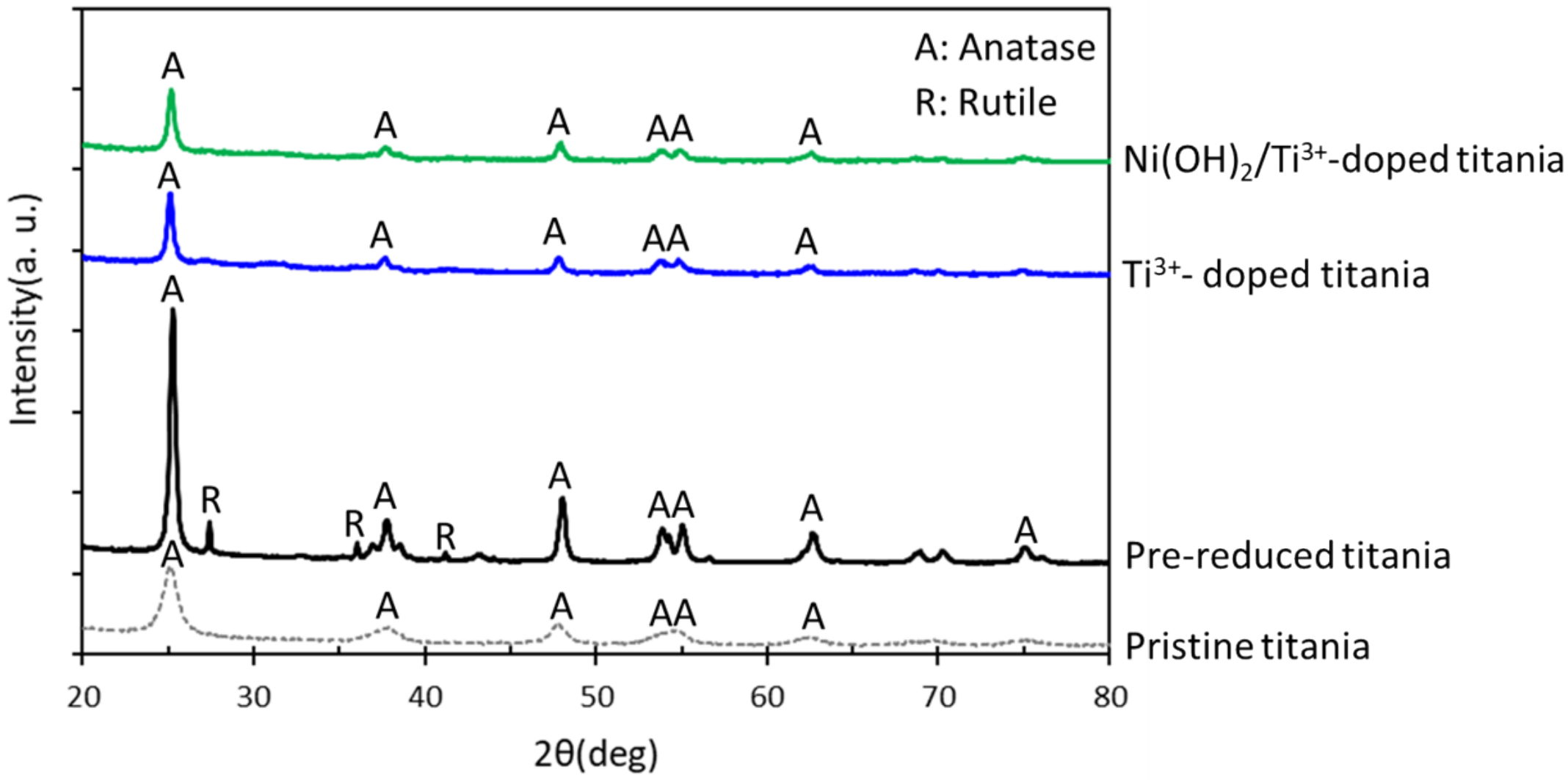

Figure S4. XRD patterns of the samples

(5) Absorption spectra and visible light responsiveness (under UV light irradiation)

Figure S5 shows the absorption spectra of the samples. The pristine titania exhibits absorption almost exclusively in the ultraviolet region, whereas the Ti^{3+}-doped titania shows visible-light absorption attributed to oxygen vacancies and Ti^{3+} species. Addition of $Ni(OH)_2$ slightly increased absorption in the visible region. When visible light (L42 cutoff filter) was irradiated concurrently with UV light, the rate of hydrogenproduction was approximately 2.7 times that obtained under UV irradiation alone, demonstrating visible-light responsiveness under UV-irradiation conditions.

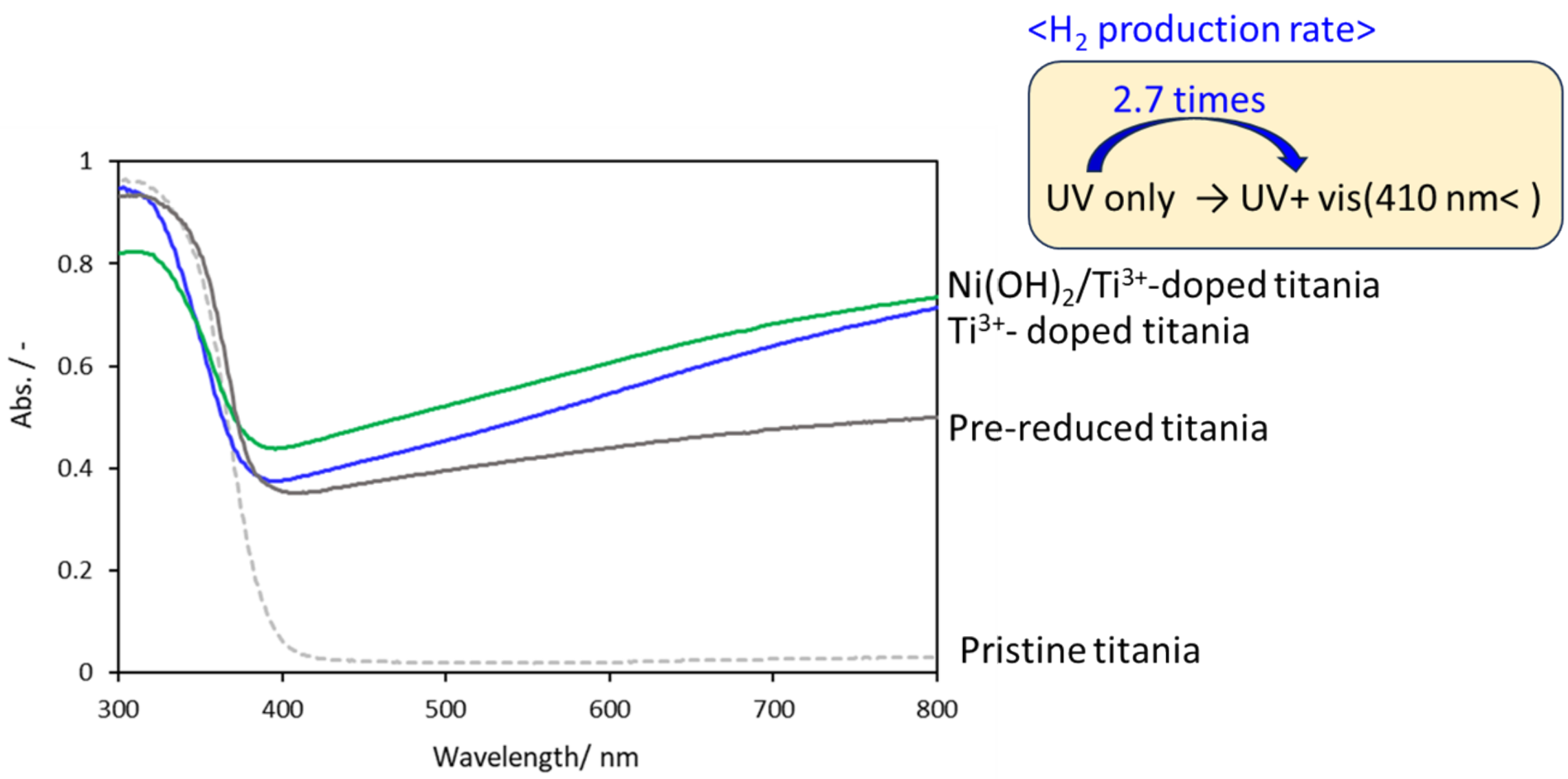

Figure S5. Absorption spectra of the samples

(6) XPS analysis

XPS analysis comparing samples after wet bead milling with pristine titania confirmed the existence of Ti^{3+}. Based on binding energies of Ti^{3+} and Ti^{4+} in the Ti 2p XPS spectra [2)], peak deconvolution was performed. The Ti^{3+} content ratio was calculated using the areas of Ti^{3+} and Ti^{4+} and the following formula: $Ti^{3+}/(Ti^{3+} + Ti^{4+})$. Pristine Titania exhibited no detectable Ti^{3+}, whereas the bead-milled sample showed a $Ti^{3+}/(Ti^{3+} + Ti^{4+})$ ratio of approximately 16%. The analysis of O1s was done in the same way.

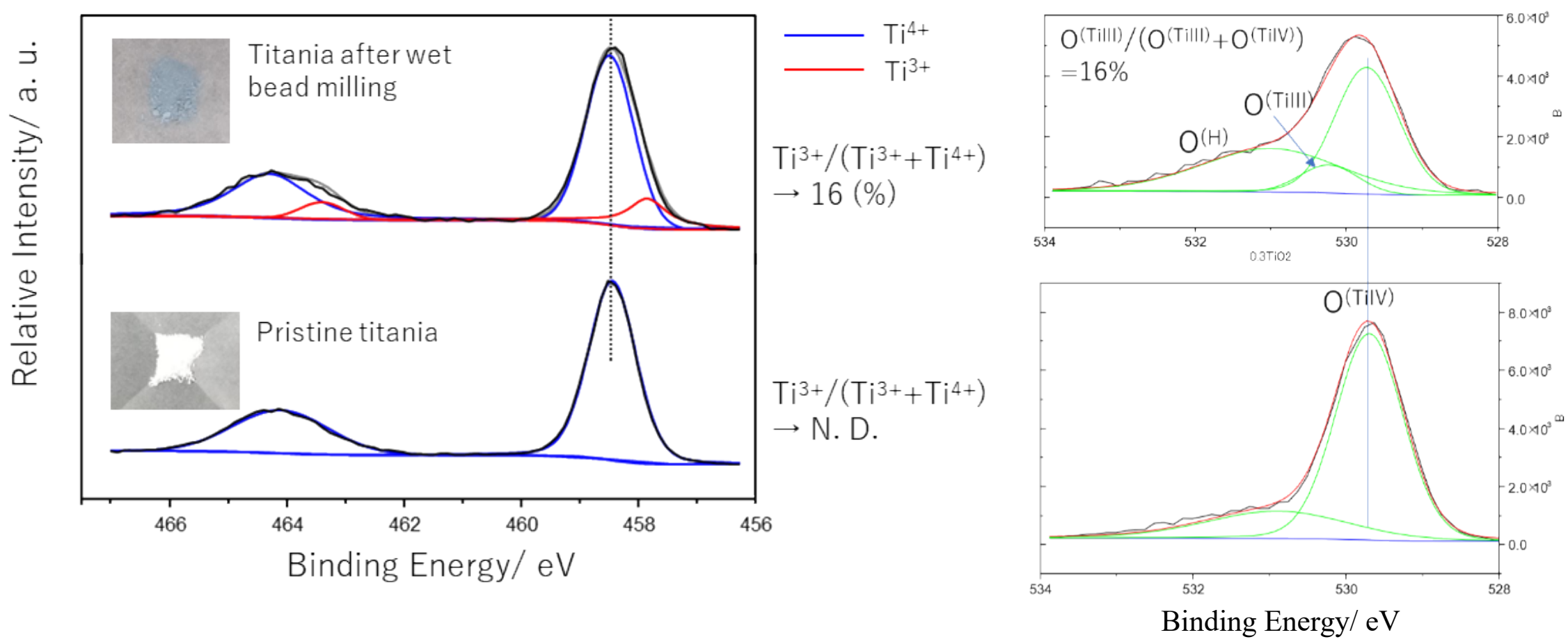

Figure S6 XPS spectra component (Ti^{3+} and Ti^{4+}) analysis of the Ti 2p peaks, and O 1s peaks

(7) Effects of the amount of concentrated artificial seawater added and the ethanol concentration

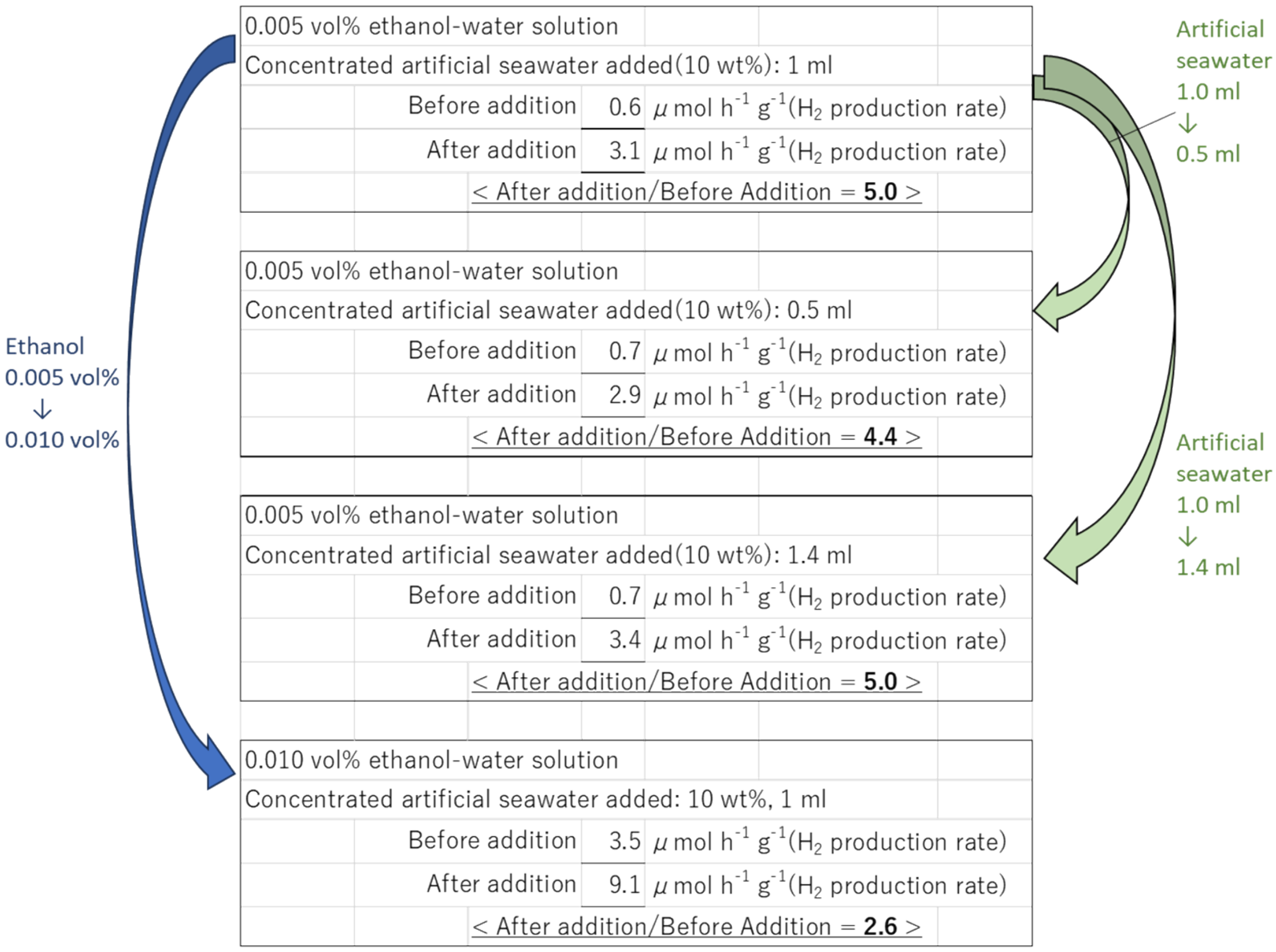

Figure S7 Effects of the amount of concentrated artificial seawater added (1.0 ml, 0.5 ml, 1.4 ml) and trace ethanol concentration (0.005 vol%, 0.010 vol%) on the hydrogen generation rate.

When the amount of concentrated artificial seawater was decreased from 1.0 mL to 0.5 mL, the fold-increase in the hydrogen evolution rate slightly decreased from 5.0 to 4.4. Increasing the amount from 1.0 mL to 1.4 mL resulted in almost no change, with the enhancement remaining around 5.0-fold. These results indicate that the activity enhancement arises from both the sacrificial oxidation of chloride ions and the electrolytes supplied by the concentrated artificial seawater (as well as trace ethanol). They also suggest that chloride ions are present in sufficient excess and are therefore not rate-limiting. In addition, when the ethanol concentration was increased from 0.005 vol% to 0.01 vol%, the total amount of hydrogen produced increased; however, the fold-increase before and after seawater addition decreased from 5.0 to 2.6. This suggests that, at higher ethanol concentrations, a larger fraction of the active sites on $Ni(OH)_2/Ti^{3+}$-titania is consumed by ethanol oxidation, which in turn diminishes the promotional effect of the concentrated artificial seawater.

(8) Comparison of the XPS spectra (Ti and Ni) of the samples before and after the reaction (Figure 4 in the main text)

XPS measurements of Ti and Ni were conducted on the sample before and after the seawater-splitting experiment (Figure 4). The results are presented in Figure S8, confirming that there were almost no changes in either the approximate amount of Ti^{3+} or the chemical state of Ni.

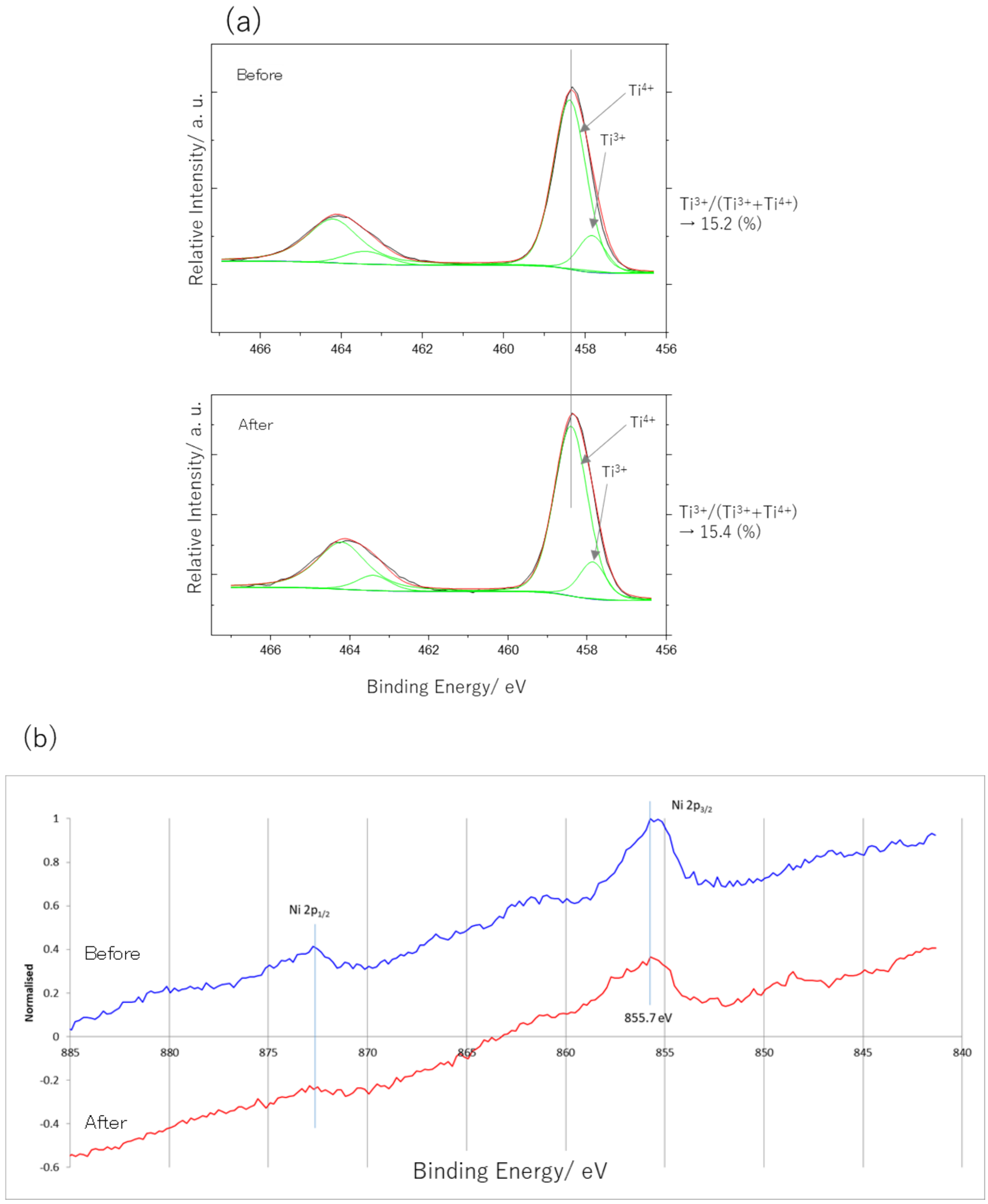

Figure S8. XPS spectral deconvolution of (a) the Ti 2p peaks (Ti^{3+} and Ti^{4+} components) and (b) the Ni 2p peaks. [Reaction conditions for Figure 4 in the main text: 0.005 vol% ethanol, 1 mL of concentrated artificial seawater (10 wt%), UV–vis irradiation, 5 h reaction.]

(9) Addition of 10 wt% Na_2SO_4 solution instead of 10 wt% artificial seawater

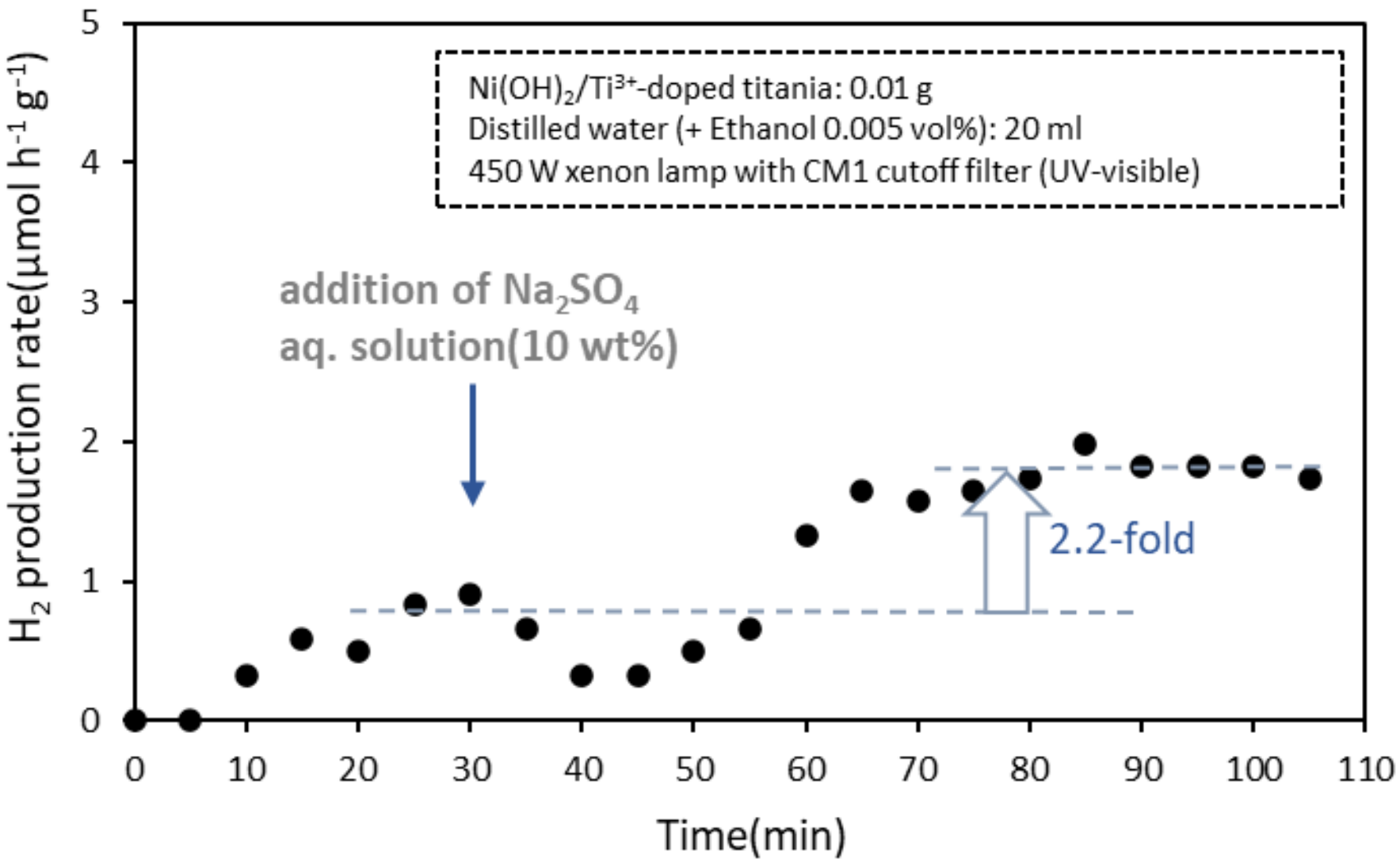

Figure S9 Effect of adding Na_2SO_4 aqueous solution (10 wt%) with traces amount of ethanol (0.005 vol%) on hydrogen production rate. (Experimental conditions: $Ni(OH)_2/Ti^{3+}$-doped titania 0.01 g, distilled water 20 ml, 450 W xenon lamp with CM1 cutoff filter.)

Hydrogen evolution increased by approximately 2.2-fold, suggesting that the electrolyte enhanced the hydrogen-generation capability of the catalyst. The promotional effect observed upon the addition of artificial seawater is therefore attributed to the combined contributions of this electrolyte-induced activation of the catalyst itself (ex. by affecting the electrical double layer and band bending on the titania surface) and the oxidation of chloride ions.

(10) Application to real seawater splitting in Tokyo Bay

The hydrogen generation reaction by seawater decomposition was verified using actual seawater from Tokyo Bay (actual seawater is thought to contain not only the components found in artificial seawater, but also dead microorganisms, oil, etc.). The results are shown in Fig. S7. When $Ni(OH)_2$ composite Ti^{3+}-introduced oxygen-deficient titanium dioxide was reacted with the addition of approximately 0.02% ethanol, hydrogen was stably generated over a long period of time, and free chlorine of approximately 1 ppm was detected (*residual free chlorine concentration is unstable).

The reaction rate remained substantially constant, and no decrease in catalytic activity was observed under the conditions.

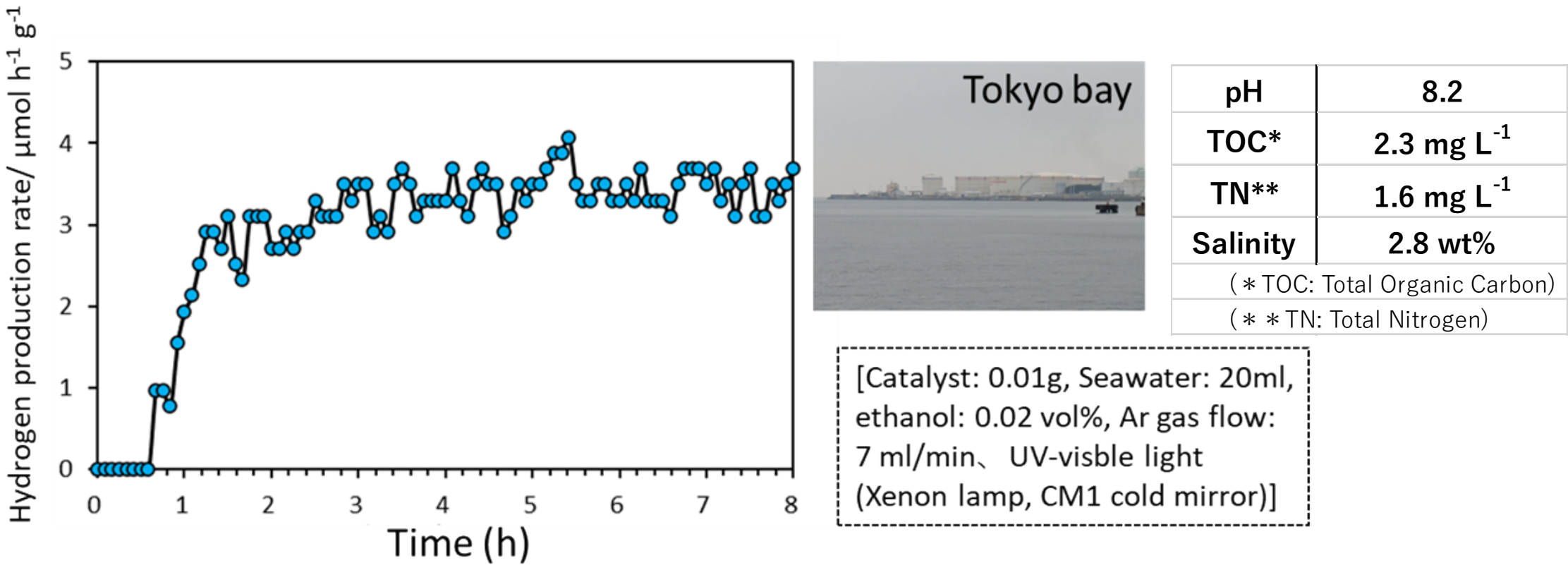

Figure S10 Hydrogen production experiment using seawater from Tokyo Bay. (Insert in upper right: Data of the Tokyo Bay seawater used.)

(11) References

1) B. S. Brunauer, P. H. Emmett and E. Teller, *J. Am. Chem. Soc.*, 1938, **60**, 309-319.

2) R. Nawaz, C. F. Kait, H. Y. Chia, M. H. Isa, L. W. Huei, N. T. Sahrin and N. Khan, *Surfaces and Interfaces*, 2022, **32**, 102146.
